# Transfer and functional consequences of dietary microRNAs in vertebrates: Concepts in search of corroboration: Negative results challenge the hypothesis that dietary xenomiRs cross the gut and regulate genes in ingesting vertebrates, but important questions persist

**DOI:** 10.1002/bies.201300150

**Published:** 2014-01-16

**Authors:** Kenneth W Witwer, Kendal D Hirschi

**Affiliations:** 1) Department of Molecular and Comparative Pathobiology, The Johns Hopkins UniversityBaltimore, MD, USA; 2) Department of Pediatrics, USDA/ARS Children's Nutrition Research Center, Baylor College of MedicineHouston, TX, USA

**Keywords:** cholesterol, diet, epigenetics, exosome, microRNA, nutrition, RNAi

## Abstract

If validated, diet-derived foreign microRNA absorption and function in consuming vertebrates would drastically alter our understanding of nutrition and ecology. RNA interference (RNAi) mechanisms of *Caenorhabditis elegans* are enhanced by uptake of environmental RNA and amplification and systemic distribution of RNAi effectors. Therapeutic exploitation of RNAi in treating human disease is difficult because these accessory processes are absent or diminished in most animals. A recent report challenged multiple paradigms, suggesting that ingested microRNAs (miRNAs) are transferred to blood, accumulate in tissues, and exert canonical regulation of endogenous transcripts. Independent replication of these findings has been elusive, and multiple disconfirmatory findings have been published. In the face of mounting negative results, any additional positive reports must provide the proverbial “extraordinary proof” to support such claims. In this article, we review the evidence for and against a significant role for dietary miRNAs in influencing gene expression, and make recommendations for future studies.

## Introduction

Eating is an engagement with the world [1] that transforms our environment into our bodies. While epidemiology studies have shown that plant-based diets help lower the risk of many diseases, synthetic supplements of vitamins and phytochemicals often do not have the same potency as complex plant materials. The lack of mechanistic understanding of the demonstrated health effects of plant-rich diets has hampered effective exploitation of this benefit. Conflicting nutritional information abounds, and popular communication of scientific studies often misinforms dietary perceptions and practices. A large branch of nutrition is based on observational studies where startling correlations can make media headlines; however, the claims often fail to be repeatable, and the published corrections appear only in the scientific literature [2]. The confused public is thus left remembering the story of women who eat additional breakfast cereal giving birth to more boys [2], for example.

A recent experimental study reported that microRNAs (miRNAs) from plants may control target genes in the consumer [3], making the same sort of media “splash” that has traditionally been reserved for observational nutritional studies. The idea that nutrition may now encompass the ingestion of genetic information is fascinating as part of the broader concept of “social RNA” presented by Sarkies and Miska in a recent *Science* editorial [4]. However, the theme of that article, much like the theme here, is that careful replication of these striking results is necessary. Emerging evidence now suggests that the initial claims of delivery and effect of foreign dietary genetic information in mammals may prove to be overstated. Nonetheless, the scientific community must be careful not to dismiss prematurely the concept of dietary transfer of genetic information. Entry of ingested miRNA into the body's pre-existing RNA-mediated regulatory pathways may prove to be specific to the genotype, dietary practices, and health status of the consumer and to the specific amount and varieties of foods ingested.

## RNA interference: Nematodes to humans

Realizing the tantalizing prospect of therapeutic RNA interference (RNAi) in humans has been a challenge in part because the confluence of three biological characteristics that facilitate RNA silencing in the nematode worm, *Caenorhabditis elegans*, is largely absent in other organisms, including closely related nematodes [5]. RNAi itself was discovered in *C. elegans* [6], and the existence of well-conserved eukaryotic silencing pathways was quickly established. Similar yet distinct systems are known in archaea and bacteria [7]. Eukaryotic RNAi is mediated by several RNA classes: miRNA, double stranded RNA (dsRNA), and, in the germ line, PIWI-interacting RNA (piRNA). At their mechanistic heart, different types of RNAi share common themes: processing of double stranded or structured, single-stranded RNA into short, single strands (usually <30 nucleotides in length); incorporation into protein machinery; binding to sequences in target messenger RNAs; and degradation, translational blocking, or sequestering of the target [8].

### How prevalent is RNAi spread in animal species?

**Figure 1:**
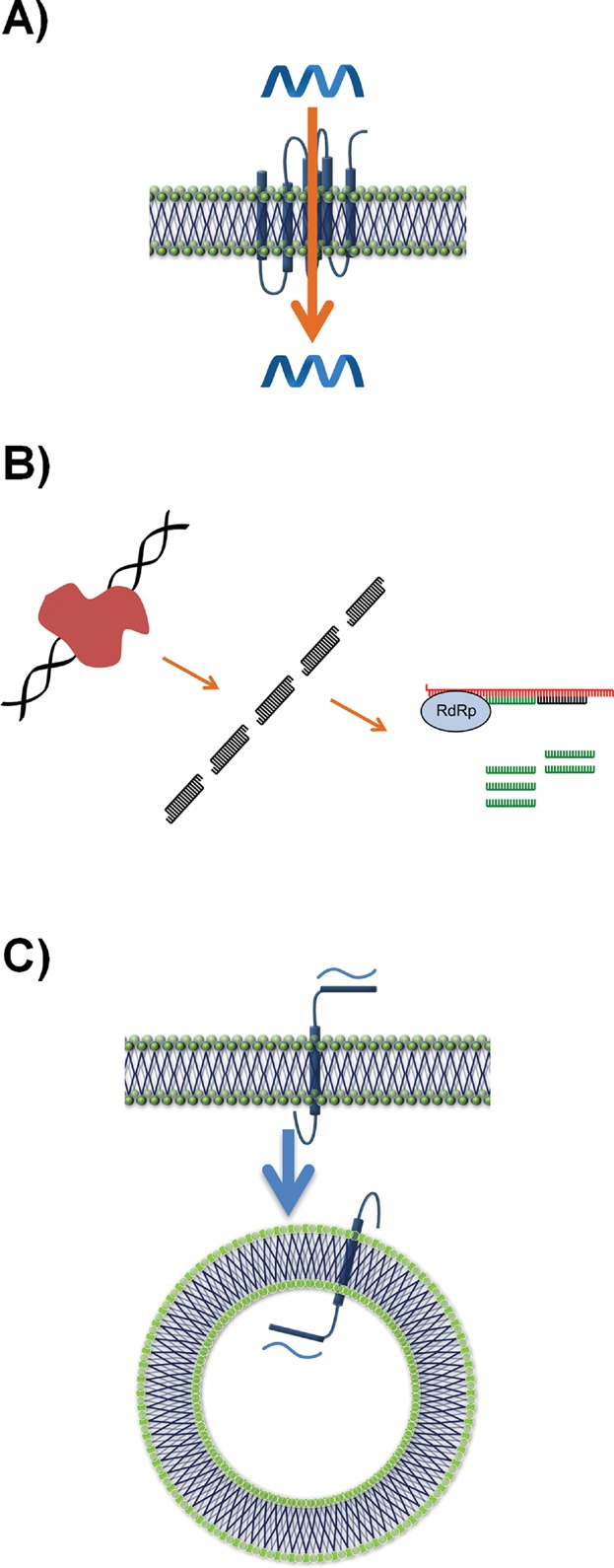
RNAi-facilitating features of *C. elegans*. **A:** Uptake of dsRNA from the environment is allowed in part by the action of transmembrane protein SID-1, promoting endocytosis of bound RNA. **B:** RNAi amplification. dsRNA molecules are processed to short “primary” siRNA (left). When an siRNA molecule binds to a target, additional target-complementary siRNAs can be generated after extension by RNA-dependent RNA polymerase and processing. **C:** SID-2 binds long dsRNA for endocytosis and secondary uptake into the cytoplasm by SID-1 (not shown). Note that cartoons are for illustration only and are not meant to represent relative size or molecular topology.

In *C. elegans*, long dsRNA injected into one part of the organism exert silencing effects in remote locations [9]. A transmembrane protein channel for dsRNA (Fig. 1A), dubbed SID-1 after a ‘systemic RNAi-deficient’ genomic locus identified in screens, is responsible [10–12]. Conservation of this channel indicated the possibility of systemic dissemination of dsRNA in many other organisms [11]. Some form of systemic RNAi exists in organisms ranging from the honeybee [13] and the flour beetle [14] to planarian flatworms [15]. However, long-range RNA transfer does not occur in the same way in vertebrates, which have developed mechanisms to recognize both single- and double-stranded RNA and its features, thereby triggering powerful innate immune responses [16–21]. Even in *C. elegans*, dsRNA distribution may not apply equally to RNA molecules of different size or type, as dsRNA of 100 nucleotides or longer are the best silencing effectors [22,23]. This is due in part to preferential uptake of longer double-stranded molecules by SID-1 [24]. Transfer of double-stranded precursors by SID-1 may, however, result in cytoplasmic presence of single-stranded RNA species [12].

### Does RNA spread occur in eukaryotes with relatively complex body plans?

A different sort of extracellular RNA (exRNA)-mediated communication may exist in some eukaryotes [25–30]. RNA molecules associate with several extracellular carriers, including lipoproteins [31] and protein complexes [32–34], but extracellular vesicles (EV, encompassing the various definitions of exosome, microvesicle, etc.) have been studied most closely. Much exRNA research has focused on miRNAs. Other RNA molecules are also found outside the cell. The similarities between enveloped viruses and EV have been examined previously [35–37], from biogenesis to cargo to information transfer capabilities.

Nevertheless, the details of how exRNA is transferred in vertebrates between extracellular carrier and recipient cell remain largely unclear. One possible mediator is a human ortholog of *C. elegans* SID-1, SIDT1 [38]. This transmembrane protein has been reported to facilitate uptake of cholesterol-modified siRNA [39] or contact-dependent transfer of human miR-21 between cultured cancer cells [40]. We expect that ongoing SIDT1 research will identify substrate specificities and its contribution to general RNA uptake or cell contact-independent transfer of RNA. A distinction should of course be made between transfer of exRNA *into* a recipient cell – with retention of function such as miRNA-mediated regulation – and stimulation of signaling from outside the cell by exRNA. In addition to recognition of dsRNA molecules by TLR3, toll-like receptors can sense short single-stranded RNA molecules [41]. Activation of TLR7 and TLR8 by specific miRNAs has been reported [42,43]. In summary, many questions remain about systemic RNAi in eukaryotes with different body plan complexity. The various proposed systems, while of considerable potential importance, are not as robust as the dsRNA-based system in *C. elegans*.

### RNAi amplification occurs in few animals

A second *C. elegans* characteristic contributing to the remarkable implementation of RNAi is amplification of RNAi effectors [44]. Secondary RNA is produced when primary siRNA, processed from long dsRNA, primes RNA-dependent RNA polymerase-mediated copying of target RNA molecules. The resulting dsRNA is then processed into secondary siRNA (Fig. 1B). Successful amplification in *C. elegans* is generally dependent on the products of the rde-1 and rde-4 genes [45]. Although amplification activity has been reported in some insects, the underlying mechanisms are not well understood, as insects lack RNA-dependent RNA polymerases [46,47]. We are unaware of evidence that amplification of silencing RNA signals occurs in humans.

### *C. elegans* samples environmental RNA robustly

Efficient uptake of dsRNA molecules from environmental sources (Fig. 1C) also enhances RNAi in *C. elegans*. Whether soaked in a dsRNA solution [48] or fed with bacteria expressing dsRNA [49], *C. elegans* assimilate dsRNA from their surroundings, often rendering experimental injection unnecessary. In contrast with SID-1-mediated spreading of dsRNA within the organism, a second protein, SID-2, allows entry of ingested RNA [50,51]. Expressing the *C. elegans* SID-2 gene in uptake-refractory organisms, including other nematodes, permitted uptake of ingested dsRNA [50,52]. SID-2 has RNA size requirements, and RNA uptake depends on an acidic extracellular environment such as that of the gut in many organisms [51].

### Environmental sampling: Not ubiquitous, but not restricted to *C. elegans*

*C. elegans* is manifestly not the only organism that can “sample” environmental RNA, although studies in organisms without amplification mechanisms necessarily involve low copy numbers and are thus fraught with the possibility of contamination artifacts [4,53]. The exact mechanistic bases of environmental sampling in different species are incompletely understood. Unlike SID-1, the exogenous RNA transporter SID-2 is poorly conserved. In concert with a functional SID-1, SID-2 preferentially allows import of dsRNA of 50 nt or longer [51]. There is some evidence that at least one SID-2-independent RNA uptake pathway may exist in *C. elegans* [54], and it is clear that other organisms can internalize ingested dsRNA by employing endocytosis that does not require SID-2 [55]. For example, scavenger receptor-mediated endocytosis may allow uptake by specific Drosophila cells [56,57]. (Mammalian cells may also internalize dsRNA by this means, albeit for presentation to the innate immune system [58].) Uptake may also be enhanced by plant substances that modify the gut barrier [59]. The list of organisms displaying environmental RNA uptake has lengthened considerably to encompass additional nematodes [5,60,61], insects [62–65], even shrimp [66], and two species of sponge [67]. dsRNA can act as a species-specific pesticide in insects [64,68–71]. A “once-removed” environmental transfer was recently reported, in which dietary dsRNA, taken up by *Apis* (bee), was transferred to an ectoparasite with functional consequences [72].

### The challenges of oral RNA delivery to mammals

Even so, simple feeding of RNA is insufficient for uptake by some insect species, including *Drosophila* [64], which lacks SID-1 transport [10,73]; this is certainly the case in mammals. It was discovered early during mammalian work that siRNA must be chemically stabilized and conjugated to ensure activity following delivery [74]. Formulation of RNAi therapeutics has since busied many investigators, and the consensus is that engineered modifications and/or “supramolecular assemblies” are essential [75] for stability and uptake, even for delivery by injection. Oral delivery remains a “holy grail.” Researchers have reported dietary delivery of RNA or other molecules, encapsulated, for example, in various polysaccharide shells [76,77] or in tumor cell-derived exosomes [78] or other vesicles [79,80]. Although uptake of orally delivered RNA may be mediated by surveilling immune cells in the gut – raising the possibility that egressing cells could transfer their exogenous cargo to distant recipient cells – RNA delivery to the gut may allow mostly local rather than systemic dissemination [81]. As a result, systemic delivery of RNAi mediators has been achieved almost exclusively by injection in mammalian studies.

## Dietary RNA transfer to vertebrates: The evidence

Because of the failure – after more than a decade of intensive inquest – to find robust mammalian homologs of *C. elegans* RNAi accessory mechanisms or to develop facile oral delivery methods for RNAi [82], a report published online in late 2011 took the scientific community by surprise. Writing in *Cell Research*, a team led by Chen-Yu Zhang described a series of striking findings on “cross-kingdom” functional RNA transfer. In this study, Zhang et al. [3] used sensitive high-throughput sequencing (HTS) methods to detect high levels of plant miRNAs, or “xenomiRs”, in the serum and/or tissues of humans, mice, and calves with plant-based diets.

### Abundance and function of xenomiRs – Comparable to those of endogenous RNAs?

At least four central findings of this work seemed poised to overturn prevailing RNAi paradigms: (i) Food-derived plant miRNAs entered mammalian circulation naturally and achieved levels comparable to those of abundant endogenous extracellular miRNAs. (ii) Plant miRNAs were detected not only in circulation, but also in all murine tissue types examined, and at copy numbers rivaling those of endogenous miRNAs. In liver, for example, some plant miRNAs were more abundant than let-7a. (iii) In the mouse, plant miRNAs were said to downregulate at least one endogenous target in liver, the low-density lipoprotein receptor adapter protein 1 (LDLRAP1), within hours of dietary intake. Interestingly, LDLRAP1 downregulation was also reported to increase circulating LDL. (iv) Perhaps most unexpectedly, uptake from the diet was independent of chemical modifications, engineered delivery vehicles, or even vesicles or other protecting carriers in the food, as chemically synthesized oligonucleotides with the same sequence as plant MIR168a were reportedly taken up with similar results.

### An attractive hypothesis lacks independently corroborating data

Dietary miRNA transfer thus appeared to be an attractive candidate to explain several unsolved mysteries in nutritional science. Observational studies have suggested that specific nutrients contribute to the observed benefits of plant food consumption, but randomized controlled trials have failed to support these conclusions [83]. Could a relationship have been missed because these studies failed to consider miRNAs as a nutrient? There is also indirect support for the concept. For example, although absorption of miRNA is not well-characterized in mammals [84], bacterial factors may facilitate uptake of RNA in the gut [85]. The possibility has also been raised that RNA in a mother's breast milk may survive the infant stomach and intestinal tract to help with gene regulation while the immune and metabolic systems are immature [86–89]. The existence of RNA in vesicles within breast milk is well-established, as is the uptake of such vesicles by cells [89]. The potential involvement of breast milk miRNAs in regulatory pathways of relevance to human infants, such as metabolism of the mother's high fat diet, has been inferred from pathway analyses [86]. However, direct evidence of functional transfer of miRNA between vertebrates – even individuals of the same species – is lacking.

Response to the *Cell Research* publication ranged from enthusiastic optimism to incredulity. Coverage in journals and the popular press included a “you are what you eat” theme and explored implications of interspecies and interkingdom information exchange. Another leitmotif was dietary miRNA as a new type of nutrient [90], one that might explain the effects of certain folk remedies [91]. Debate about the findings also became a surrogate for genetically modified organism (GMO) controversies. There were suggestions that Zhang's results underscored concerns about unintended RNA-mediated effects of GMOs [91]. We note, however, that these latter two issues were not directly addressed in Zhang's studies or, as far as we know, in any subsequent publications. Indeed, little evidence to support the findings has emerged in the two years, whether from the Zhang lab or from independent groups.

## Questions and counterevidence

Amidst intense specialist and lay interest in the Zhang et al. findings, questions have been raised about systemic distribution of xenomiRs following dietary intake and about the likelihood of post-transcriptional regulation. These questions have stemmed from critical review of the initial publication and examinations of related studies. Importantly, numerous negative experimental studies, including several initiated expressly to confirm the findings of Zhang et al. [3], have now been published [82,92,93].

### Critique of published positive findings

Several features of the initial, positive publication have been critiqued [53,94]. Only four plant miRNAs were consistently detected in humans, and with puzzling variability. Baseline and post-feeding levels of two xenomiRs in vertebrate blood and tissues reportedly approximated the abundance of moderately expressed endogenous miRNAs. If endogenous miRNAs regulate transcripts, as is widely assumed, exogenous RNAs could achieve similar effects. However, the minimal apparent changes in xenomiR levels following feeding are difficult to reconcile with rapid, detectable downregulation of mammalian targets. Finally, the levels of reported exposure in the original experiments would be difficult to replicate in humans. These three critiques are presented in greater depth in Box 1.

**Box 1 Critical observations on Zhang et al**

**Donor pool variability suggests non-dietary explanations**

**Figure 2:**
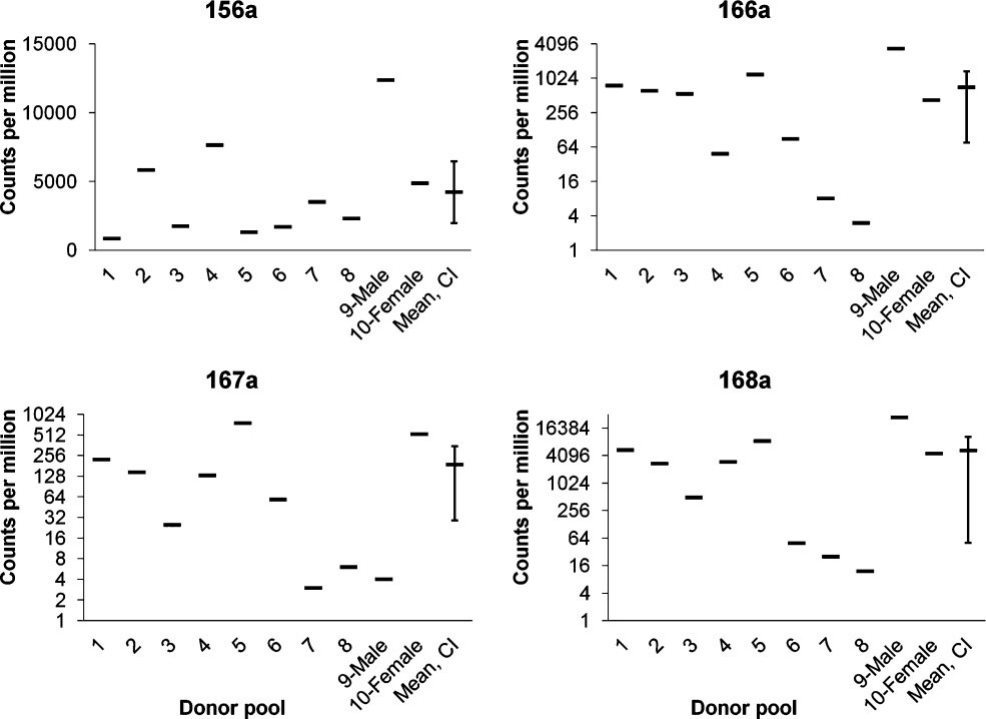
Variability of apparent xenomiR detection in human sera: reanalysis of Supplementary Data from Zhang et al. Four plant miRNAs were detected in each of 10 human serum pools examined in the original dietary miRNA study [3]. Pools were composed of 10–11 individuals each, with no assignment criteria specified (other than gender for the two indicated pools). There was substantial variability between pools. Shown are reads per million animal miRNAs for each pool, followed on the right of each panel by a 95% confidence interval for all miRNA-specific data.

Pooling samples can be an effective strategy in certain circumstances but will also mask biological variability. In Zhang et al., though, plant miRNA reads in ten serum pools derived from 10 to 11 donors each varied strikingly [3,53] (Fig. 2). MIR168a reads varied >2,000-fold. The necessarily even more profound variability at the sample level does not seem reconcilable with a general biological phenomenon and would be more easily explained by other factors:

*A minority of individuals were responsible for all or most pooled reads*. As a corollary, most donors may not have had detectable plant miRNAs in serum.

*Several donor pools represented distinct yet remarkably uniform populations*. XenomiRs were detected at low frequency in donor pools 7 and 8, such that just one donor with detected miRNAs around the population average would have resulted in an upward revision of the pooled average by an order of magnitude or more. However, this presupposes non-random pooling; other than the male and female pools, there was no indication of pooling based on distinct population characteristics.

*Technical variability resulting from RNA extraction, sequencing, or library construction*. Variability of RNA extraction, even in replicate purifications, can be large, especially with technically challenging non-kit-based methods [96]. Technical HTS replicates may be unnecessary, but greater variability will result from library construction from the same source material, distinct purifications of the same source material, and, of course, biological replicates. Since many HTS studies have relied on only one or two samples per biological group [97], perhaps the reported variability is largely technical.

*Contamination and batch effects*. Contamination by sources such as oligonucleotide standards and non-dietary environmental plant matter presents a risk of false positives. Pollen contains miRNAs [92,98,99] and may be introduced at any stage of sample collection and preparation. Batch effects may arise from collection, storage, purification, and experimental factors. Seasons, geography, and differences in air filtration between collection and laboratory facilities could explain why certain batches of human samples might artifactually display drastically different levels of “circulating” plant miRNAs.

**Stoichiometry and timing of regulation**

Three hours after feeding, there was no significant difference in plasma or liver levels of MIR168a between mice fed regular chow or raw rice. By 6 hours, a 50% increase in serum and a twofold increase in liver were observed, accompanied by >50% decrease of putative target LDLRAP1. Such a striking, rapid downregulation requires a confluence of several factors. First, a fold change in serum MIR168a within the usual margin of error for qPCR assays would have to result, on average, in a doubling of copy numbers in liver cells in less than 3 hours. Second, baseline suppression of the LDLRAP1 transcript would have to reach near 100% suppression despite the modest fold-change in miRNA. (Reporter assays used to confirm miRNA-mediated regulation through the 3′ UTR may require thousands-fold changes in the levels of targeting miRNAs to obtain observable regulation over 24–48 hours or more.) Third, even in the unlikely event of immediate, 100% suppression of LDLRAP1 translation, the extant protein would have to have an extremely short half-life to experience >50% reduction in less than 3 hours. It is uncertain whether or not these prerequisites are fulfilled for MIR168a and LDLRAP1. With small fold changes derived from a semi-quantitative Western blot [3] for LDLRAP1, clarifications and independent confirmation of these remarkable results might be useful.

**Relevance of intake level and nutritional value**

To approximate the exposure of mice on a raw rice diet [3], a 55 kg human would have to eat 33 kg of cooked rice per day [94]; the apparent regulation of LDLRAP1 (and, by extension, cholesterol) observed in mice might not occur in humans. The fact that the rural Chinese population consumes predominately rice-based diets yet have lower levels of LDL than their urban counterparts (with lower rice intake) suggests that the proposed cholesterol-regulating activity of MIR168a in humans is negligible compared with the many known regulatory mechanisms. Indeed, the cholesterol results are more simply explained by the nutritional insufficiency of a raw rice diet, as recently confirmed [82].

### Public data: Consistent with artifact or scant uptake of dietary RNA

Of 83 public HTS datasets analyzed for evidence of plant miRNAs in animal fluids and tissues [95], 53 yielded reads from fewer than three apparent plant miRNA species. MIR156a was detected among the top three plant miRNA sequences in 18 of 83 profiling studies (22%), or 13 of 76 non-insect studies (17%), albeit with median counts of less than six per million animal miRNA reads (range: 0.4–1,089). MIR168a was registered somewhat more often, at a median of 181 counts per million animal miRNA reads in 37 of 76 non-insect studies (49%). In contrast, both MIR156a and MIR168a read counts in the Zhang study were above 2,800 per million in some sample pools [3]. In several reviewed datasets, a MIR168 variant was detected in animals that did not receive food containing that miRNA [95]. This observation and a general discrepancy between apparent miRNA abundance in food source and ingesting animal led the authors to conclude that MIR168 appears to be a special case: reported sequencing reads for MIR168 in vertebrate studies might be largely artifactual.

### Negative studies

Independent studies have now presented data that are difficult to reconcile with general uptake of exogenous miRNAs at the levels reported by Zhang et al., levels that might be consistent with regulatory effects.

#### Negative results following feeding in bees, mice, and humans

In 2013, Snow et al. [92] reported in *RNA Biology* that plant miRNAs were undetectable or indistinguishable from background signal in experiments conducted with three organisms: humans, mice, and bees. In plasma of human athletes with diets that included fresh fruits, plant miRNAs could not be detected. Bees also did not appear to take up plant miRNAs.

Wild type mice with diets containing very different levels of specific miRNAs did not have above-background levels of plant miRNAs in tissues [92]. Although a slightly higher signal for plant miRNA MIR156a was observed in plasma of mice with plant- versus lard-based diet, levels in the latter were at the limit of detection, and there were no significant differences. A fresh avocado diet did not result in significant increases of plant miRNA. The authors concluded that plant miRNAs – if present at all in the ingesting animal – were found at less than one copy per cell even in presumed target organs such as liver and kidney. In a particularly elegant and definitive experiment, miR-21 knockout mice that have no detectable miR-21 expression [100] were fed for four weeks with an animal lard diet containing high levels of miR-21. At the end of four weeks, no miR-21 was detected above the background level of the qPCR assay in plasma or in any of four tissues (lung, liver, kidney, and stomach) [92].

#### Non-human primate time-course: Negative

A smaller study, also published in *RNA Biology*, arrived at similar conclusions [93]. Blood was drawn from two non-human primates before and at 1, 4, and 12 hours following ingestion of a miRNA-rich soy and fruit mixture. At- or near-background levels of several plant miRNAs were recorded by qPCR assay, but there was no indication of response to feeding. Importantly, from the results of digital droplet PCR experiments, the authors inferred that much or all of the low-level signal they observed was due to non-specific amplification.

#### Negative results of a direct replication study

The *RNA Biology* studies did not repeat the Zhang et al. mouse experiments or examine LDLRAP1, the putative target of miR168a, but a comprehensive attempt by Monsanto and Miragen researchers to replicate the findings was published several months later in *Nature Biotechnology* [82]. In this study, groups of mice received one of three dietary formulations: standard processed chow, a nutritionally sufficient diet containing 41% rice, or raw rice with a binding agent. This was an improvement on the original study design, which could not address the question of whether dietary insufficiency (raw rice) or LDLRAP1 downregulation by miRNA precipitated the reported LDL increase (see Box 1). Dickinson et al. showed by qPCR that even the 41% rice diet contained more MIR168a than was administered by Zhang et al., likely due to rice strain differences. LDL levels actually fell in this group, but rose in the all-rice group. The parsimonious explanation is that nutritional intake, not xenomiRs, increases LDL as the body mobilizes energy reserves. Importantly, levels of LDLRAP1 were unchanged across groups, as measured with a sensitive and quantitative ELISA assay. Little or no plant miRNA was found in blood or organs of mice fed with any of these diets, as measured by both HTS and qPCR [82].

#### Negative results in humans and mice with liver damage

In late 2012, detection of numerous exogenous RNA sequences in human circulation was reported [101]. However, these included only one plant miRNA, MIR168, the same xenomiR that is curiously and perhaps artifactually overrepresented in public HTS datasets [95]. Furthermore, this study found MIR168 at only single digit copies per million reads [101], orders of magnitude below the originally reported levels [3]. This detection level and the identity of the plant miRNA should rightly be viewed as disconfirmatory of Zhang et al.

In a second study by some of the same researchers, blood samples were taken from mice that did or did not receive liver-damaging doses of acetaminophen [102]. Exogenous RNAs of various types were reportedly detected in blood, including sequences from plants to which the animals do not seem to have been exposed. Only one plant miRNA was found consistently, but, again, it was MIR168a, mapped at fractional reads per million. XenomiRs from various insect and worm species were also detected, albeit in only one or two samples and at similarly low levels. Several of these sequences have been found in organisms to which the mice probably would not have been exposed, are found at low abundance endogenously, or mapped to rare processing variants, not the canonical miRbase sequence of the identified miRNA (Witwer, unpublished analysis of data from [102]). While interesting and actually presented as confirmatory of Zhang et al., these findings are likely the result of noise in sequencing and analysis, not low-level biological uptake of trace food contaminants. In contrast with the HTS data analyzed in *BMC Genomics* [95] or provided by Dickinson et al. [82], but similar to the original work [3], the data underlying these studies do not seem to have been made available in public archives.

## Paradigms and hypotheses

It does not appear that transfer of miRNAs from the diet is a reliable occurrence in vertebrates, or that transferred molecules are normally sufficient in number to exert regulatory pressure. Nevertheless, it remains possible that transfer happens under certain circumstances. There may be room for additional research in this area (Table 1) to address several key questions, including those outlined below. Along the way, as also highlighted here, optimized procedures are needed to balance sensitivity and specificity, and careful control experiments are important to avoid overinterpretation of positive results.

**Table 1:** Topics for further study

Requirement	Comments and proposals
Proper normalizers for exogenous RNA experiments	Endogenous miRNAs that change with the circadian day, exercise, feeding, or drugs might be inappropriate. Synthetic spike-in(s)?
Development of tissue sensors	Extremely sensitive tissue sensors (as available in *C. elegans* [11]) could identify effects of low-level RNA transfer
Understanding of uptake mechanisms	Additional work on SIDT1, scavenger receptors, and other transmembrane proteins; further exploration of extracellular vesicles and viruses
Identification of conditions, treatments that facilitate RNA transfer	Examine samples from humans with gut permeability conditions: microbial and small RNA transfer? Are there dietary or medicinal substances with unappreciated effects on permeability?
Appreciation of human/model differences	Mice and humans: humans utilize a greater proportion of calcium absorbed in the upper small intestine [125]. Might there also be differences in nucleic acid uptake? Use of humanized mouse models [126]

### How would xenomiRs cross the intestinal barrier?

#### Transcytosis and other mechanisms

**Figure 3:**
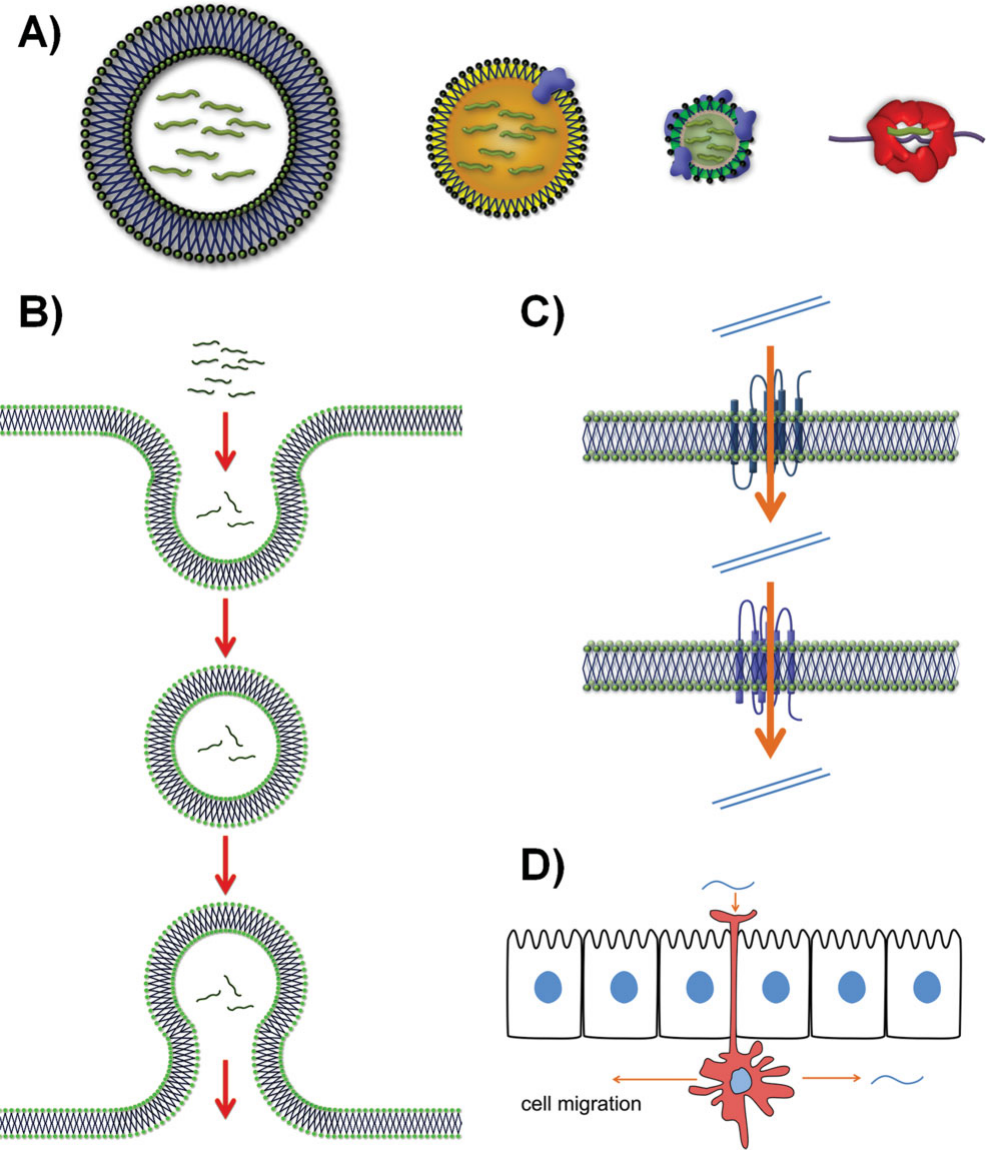
Extracellular carriers and trans-barrier transport of RNA. **A:** Extracellular RNA is carried by and protected by incorporation into extracellular vesicles (EV, left) including exosomes, microvesicles, some viruses, and other membrane-bound entities; low-density (middle left) and high-density (middle right) lipoprotein particles; and protein complexes such as Argonaute-containing structures (right). The number of RNA molecules within such carriers is not well known. **B:** Transcytosis may deliver RNA from one side of a cell barrier to the other. Although uncomplexed RNA is shown here, uptake of RNA carriers is more likely. **C:** Transfer of RNA molecules via transmembrane channels. Here, a double-stranded species is transferred. **D:** Immune cells in biological barriers may sample nucleic acids and other molecules on one side and release them on the other, with or without movement of the immune cell to a new location.

Given the instability of naked RNA, transcytosis – vesicular uptake of RNA carriers and their cargo (Fig. 3A) on one side and release on the other side of a biological barrier – is an attractive option (Fig. 3B). Alternatively, uptake into the cytoplasm by transmembrane RNA transporters is hypothetically possible (Fig. 3C) but would require close contact with a cell or carrier that releases the RNA in the immediate vicinity of the transporter. Additionally, immune cells could take up RNA/RNA carriers and release them on the other side of the barrier (Fig. 3D).

#### Sequence specificity?

Degradation-resistant RNA molecules in dietary materials would be packaged into protein complexes and/or vesicles. Since transport mechanisms would recognize the carrier, not its cargo, it is difficult to envision how hypothetical sequence specificity could be achieved at the level of uptake. Specific sequences could be incorporated into protective carriers in the source material (say, by RNA binding proteins), but it is not clear that this occurs.

#### Permeability and mass action

Every biological barrier has some degree of leakiness. With sufficient dietary intake, some foreign RNA might survive the trip to the intestine. A small number of molecules could traverse the gut and enter the bloodstream. In the absence of sequence-specific uptake, which has not been demonstrated, the transfer of foreign RNA molecules should mirror their distribution and abundance in the source material.

As we have stated, one explanation of the reported human donor pool variability would be uptake of plant xenomiRs by only a small number of individuals, perhaps as a result of altered intestinal permeability. Inflammatory states associated with infectious and non-infectious GI tract disease, stress, and malnutrition can lead to a leaky barrier, and the influences of obesity and alcohol use have also been described. Traditional folk remedies have not been well characterized and might include alteration of gut permeability. Admittedly, the work of Wang et al. [101] does not support this hypothesis: there was no enrichment of plant MIR168a in the blood of colitis patients. Nevertheless, intestinal permeability remains an area to explore.

### Are plant-specific protocols needed for detection of plant miRNAs?

2′ *O*-Methylation of plant miRNAs on the ribose of the 3′ nucleotide [103], a modification also associated with animal piRNAs [104], has been reported to interfere with T4-mediated ligation of adapters, as, for example, during HTS library preparation [105,106]. Bias could lead to underrepresentation of plant sequences in mixed plant and animal libraries. Longer ligation times, higher enzyme concentration, selection of relatively unselective enzymes, and addition of ligation enhancers such as polyethylene glycol may promote more accurate comparison of plant and animal sequence levels in such libraries [106].

While optimization of plant- and animal-specific library preparation protocols is useful, protocol differences cannot explain the discrepancies between Zhang et al. and other studies. Plant HTS libraries have demonstrably been prepared successfully using standard protocols, and any plant-specific ligation issues would simply reduce the apparent plant/animal ratios; they would not explain the near or complete absence of plant miRNAs in most datasets. There is no indication in the Zhang article [3] or papers cited in their methods section [107,108] that extraordinary steps were taken to promote plant-specific ligation. Importantly, a ligation-independent stem-loop reverse transcription qPCR method was used in three recent studies reporting negative results [82,92,93]. Dickinson et al. also performed a spike-in experiment (adding plant RNA to mouse RNA in a ratio informed by the levels reported by Zhang), and reported that their HTS results were not biased against plant miRNAs [82].

### Artifact: The challenge of sensitive methods

Too much sensitivity, rather than too little, is often a problem. The pervasiveness of nucleic acid contamination was elegantly illustrated by Charlson et al. [109]. Searching for constituents of the lung microbiome, the authors sequenced a variety of technical controls and replicates. Since many “lung” microbial species hailed from saliva contamination of the bronchoscope, the investigators refined their collection method by inserting a second sterile scope inside the first. Interestingly, microbial sequences were also recovered from sterile buffer washes of bronchoscopes: although “microbiologically sterile”, nucleic acid fragments could persist through manufacturing or sterilization processes. Sequence reads could even be obtained by purification and sequencing of sterile buffer itself. Only through careful consideration of the various sources of contamination could a true picture of the lung microbiome emerge [109]. The “de-discovery” of the xenotropic murine leukemia virus-related virus provides a second cautionary tale of contamination of various sorts [110–112]. More recently, contaminated spin columns were implicated in the “discovery” of an apparently novel parvovirus [113]. Considering the use of the Zhang paper to question the safety of RNAi technologies, it is also worth mentioning that results of transgenic DNA uptake studies have been highly variable and in some cases consistent with contamination. While some groups have reported detection of transgenes in ingesting organisms, others are unable to find even highly abundant natural food source DNA, including chloroplast genes.

Since plant biology laboratories on the thoroughly pollen-dusted surface of our planet contain many potential contaminants – natural and artificial – special care must be taken to rule out such contaminants in the case of positive results. Sequencing and amplifying technical controls to assess sequencing instrument, reagent, supply, and sample contamination may seem wasteful, but it might help to answer the many questions about positive results. In this regard, investigators would do well to borrow a page from the experience of forensic scientists, who have developed particularly rigorous procedures to reduce, if never entirely eliminate, the chance of contamination.

Finally, although the weight of the current evidence may convince many that plant RNA uptake by vertebrate animals is an infrequent event, a consortium approach may be advisable to answer the question definitively à la the XMRV Scientific Research Working Group [114]. Samples generated in or collected by a central laboratory could be blinded and distributed to several testing centers, including labs that have previously reported negative or positive results. Samples could be from a de novo feeding study, specimens with spiked-in controls, and even samples with reported positive or negative readings from one or more contributing labs. This approach would not be able to identify contamination definitively, but it might allow emergence of consensus on experimental protocols and results.

### Do low numbers of xenomiRs preclude all forms of small RNA-mediated regulation?

Non-physiologic expression of both target and putative effector RNA in cultured cells is a common approach to establishing canonical miRNA regulation through RISC. However, miRNA copy numbers in the hundreds or more per cell may be helpful but not always necessary to achieve some measure of canonical target silencing by a targeting miRNA. Mullokandov et al. [115], for example, used a series of reporters with target sites for specific miRNAs, and observed significant silencing of 67 sensors. Fifty seven of the 67 targeting miRNAs were present at 100 copies per million (0.01%) or more. This evidence has been interpreted by some to suggest that “miRNAs that represent <0.1% (sic) of (a) miRNA pool are unlikely to be functionally relevant” [116]. However, in the Mullokandov et al. study, 10 of 67 miRNAs were present at <0.01%. Combined with the observation that most examined miRNAs, abundant or not, did not suppress their cognate sensors [115], it is apparent that factors beyond miRNA copy number contribute to individual suppressive potency. Among others, subcellular localization [115] and total target numbers [117,118] influence the copy number of miRNA needed to observe an effect on any particular target. In some circumstances, this could, hypothetically, require only a few copies of a miRNA per cell.

**Figure 4:**
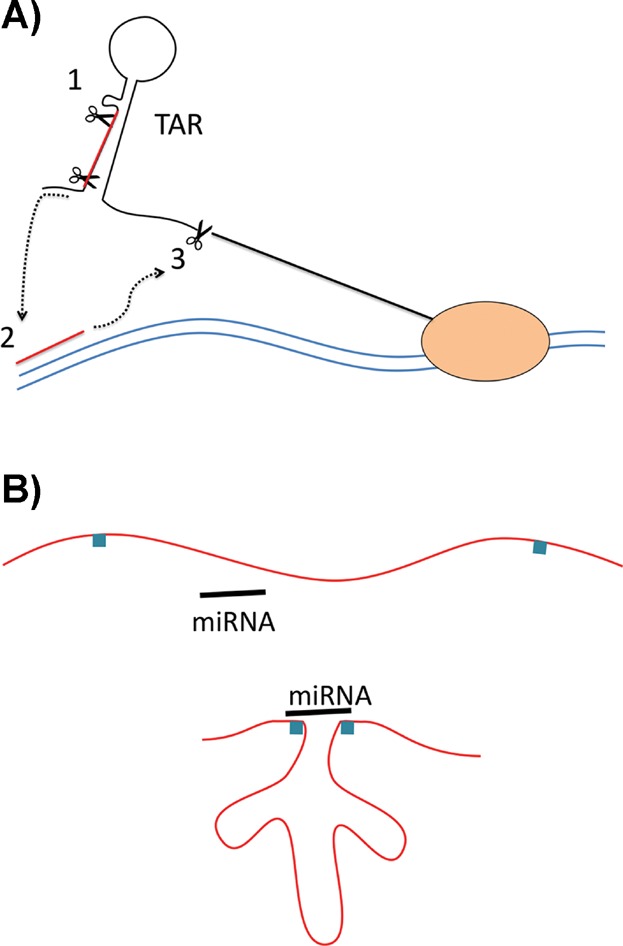
Non-canonical (RISC-independent) actions of small RNA. **A:** TAR-derived RNA in HIV-1 transcriptional control. Effects on transcription have been reported for miRNA and other small RNA. Cleavage of a 5′ structured element (TAR, 1) in the nascent HIV-1 transcript may interact with the corresponding sequence in the integrated DNA (2) and lead to further degradation of the transcript (3), enforcing HIV latency. **B:** Small RNA may influence secondary/tertiary structure by binding complementary elements separated by considerable distance in the primary sequence.

Nor is canonical RISC-mediated suppression the only conceivable or known mechanism of small RNA function. Nuclear effects of small RNA (Fig. 4A) have been reported [119], including the interference of a TAR-derived RNA with retroviral transcription [120,121]. Since only one genomic integration of HIV-1 typically occurs per cell, chromatin-mediated suppressive mechanisms might require fewer copies of suppressive small RNAs than would be needed to silence many transcripts. Indeed, this mechanism would involve, at most, one small RNA per transcriptional event (Fig. 4A). Althaus et al. [122] also reported host and viral small RNAs interactions with the HIV-1 transcript that did not necessarily involve RISC. Interestingly, the reported resistance of HIV-1 to RISC-mediated miRNA regulation [116] could theoretically be due in part to effects of small RNA, which may enforce transcript structure and transport [123,124]. Structural contributions of small RNA could of course be extended to any type of RNA molecule in the cell (Fig. 4B).

However, although it is tempting to speculate that low copy number miRNA could exert influence through canonical or non-canonical means, homeopathic effects are implausible: at least a few copies of a targeting miRNA would need to be added to a cell to achieve a change in target amount. As Snow et al. [92] observed, dietary plant miRNAs, even if reliably quantifiable, are present at less than one copy per cell in target organs. Whatever the prevalence of non-canonical miRNA-mediated regulation, a wide gulf separates the theoretical possibility of xenomiR effects at low copy numbers from the non-physiologic conditions of miRNA-target assays that are often used to establish canonical, direct regulatory effects. In one recent example, RNA species detected in blood were assessed for regulatory effects using a cell culture reporter system [101] and 10 nanomolar transfected RNA. In contrast, the overall RNA concentration in plasma was estimated to be less than 300 fM. Since the authors also estimated that the RNA they studied made up <0.01% of the total, even cells in direct contact with blood would be exposed to 300 million-fold lower concentrations of the RNA than was used in the in vitro experiments … and without the benefit of transfection reagents. Results of such experiments, while valuable, may not apply to RNA present in vivo in only trace amounts if at all.

### Intestinal permeability, intestinal deliver, and extra-dietary transfer

As we previously mentioned, intestinal permeability may be altered by certain substances, allowing measurable uptake of dietary RNA. Disease states could also affect uptake and/or clearance. In these cases, the observed effects would likely be general. Detection should closely follow RNA distribution in the dietary material, and gut bacterial products should also be detected at higher levels. Permeability and clearance aside, the results of plant vesicle administration [79,80] suggest that xenomiR presence in the intestinal epithelium might be a more appropriate subject for future exogenous miRNA studies than abundance in blood or distant organs.

Snow et al. astutely reviewed non-dietary means of systemic xenomiR transfer [92]. Indeed, organ or stem cell transplants, blood transfusions, pregnancy, and various states of parasitism may involve direct RNA transfer from one organism to another. Inhalation of atmospheric plant material, injection of plant-based drugs, and fluid exchange (e.g. through different sexual practices) could allow RNA transfer. These mechanisms might present better opportunities for study of foreign RNA-mediated regulation than low-level or undetectable systemic transfer of RNA from the diet.

## Concluding remarks

The scientific community has had two years to digest the first report that oral consumption of plant-derived miRNAs affects vertebrate metabolism. The popular and scientific press has accorded at least the requisite 15 minutes to this concept. However, as the poet Emily Dickinson wrote, “Fame is a fickle food – Upon a shifting plate.” This adage may be appropriate here, as several well-designed studies have been unable to confirm that gastrointestinal uptake is a general means of miRNA transfer and subsequent gene regulation. As the initial mixture of enthusiasm and skepticism has shifted, we must carefully consider alternative explanations for the original results. If xenomiRs as a field of inquiry are to endure, any future positive studies should rule out contamination, address rigorously the kinetics and mechanisms of dietary miRNA absorption, distribution, and clearance, and include sensitive assay systems to measure functionality in a reproducible and physiologically relevant manner for any putative diet-derived miRNA.
